# Association of Reducing the Recommended Colorectal Cancer Screening Age With Cancer Incidence, Mortality, and Costs in Canada Using OncoSim

**DOI:** 10.1001/jamaoncol.2023.2312

**Published:** 2023-07-20

**Authors:** Anastasia Kalyta, Yibing Ruan, Jennifer J. Telford, Mary A. De Vera, Stuart Peacock, Carl Brown, Fergal Donnellan, Sharlene Gill, Darren R. Brenner, Jonathan M. Loree

**Affiliations:** 1 BC Cancer, Vancouver, British Columbia, Canada; 2 Department of Cancer Epidemiology and Prevention Research, Alberta Health Services, Calgary, Canada; 3 Division of Gastroenterology, University of British Columbia, Vancouver, British Columbia, Canada; 4 Faculty of Pharmaceutical Sciences, University of British Columbia, Vancouver, British Columbia, Canada; 5 Faculty of Health Sciences, Simon Fraser University, Vancouver, British Columbia, Canada; 6 Canadian Centre for Applied Research in Cancer Control, Vancouver, British Columbia, Canada; 7 Division of General Surgery, St. Paul’s Hospital, Vancouver, British Columbia, Canada; 8 Departments of Oncology and Community Health Sciences, Cumming School of Medicine, University of Calgary, Calgary, Canada

## Abstract

**Importance:**

Recent US guideline updates have advocated for colorectal cancer (CRC) screening to begin at age 45 years in average-risk adults, whereas Canadian screening programs continue to begin screening at age 50 years. Similarities in early-onset CRC rates in Canada and the US warrant discussion of earlier screening in Canada, but there is a lack of Canadian-specific modeling data to inform this.

**Objective:**

To estimate the association of a lowered initiation age for CRC screening by biennial fecal immunochemical test (FIT) with CRC incidence, mortality, and health care system costs in Canada.

**Design, Setting, and Participants/Exposures:**

This economic evaluation computational study used microsimulation modeling via the OncoSim platform.

**Main Outcomes and Measures:**

Modeled rates of CRC incidence, mortality, and health care costs in Canadian dollars.

**Results:**

This analysis included 4 birth cohorts (1973-1977, 1978-1982, 1983-1987, and 1988-1992) representative of the Canadian population accounting for previously documented effects of increasing CRC incidence in younger birth cohorts. Screening initiation at age 45 years resulted in a net 12 188 fewer CRC cases, 5261 fewer CRC deaths, and an added 92 112 quality-adjusted life-years (QALYs) to the cohort population over a 40-year period relative to screening from age 50 years. Screening initiation at age 40 years yielded 18 135 fewer CRC cases, 7988 fewer CRC deaths, and 150 373 QALYs. The cost per QALY decreased with younger birth cohorts to a cost of $762 per QALY when Canadians born in 1988 to 1992 began screening at age 45 years or $2622 per QALY with screening initiation at age 40 years. Although costs associated with screening and resulting therapeutic interventions increased with earlier screening, the overall health care system cost of managing CRC decreased.

**Conclusions and Relevance:**

This economic evaluation study using microsimulation modeling found that earlier screening may reduce CRC disease burden and add life-years to the Canadian population at a modest cost. Guideline changes suggesting earlier CRC screening in Canada may be justified, but evaluation of the resulting effects on colonoscopy capacity is necessary.

## Key Points

**Question:**

What are the potential effects of earlier colorectal cancer (CRC) screening on disease incidence, mortality, and health care costs in Canada?

**Findings:**

This modeling study followed simulated individuals representative of the Canadian population. Beginning screening by biennial fecal immunochemical test (FIT) at age 45 or 40, rather than 50 years, was associated with decreased CRC incidence, mortality, and overall CRC-related health care costs.

**Meaning:**

Lowering the screening initiation age in Canada from the current start age of 50 years may be justified.

## Teaser

This economic evaluation computational study uses microsimulation modeling to estimate the association of a lowered initiation age for colorectal cancer screening by biennial fecal immunochemical test with colorectal cancer incidence, mortality, and health care system costs in Canada.

## Introduction

Organized colorectal cancer (CRC) screening programs exist or are planned in every Canadian province and territory, contributing to declining CRC incidence.^1^ As per 2016 Canadian Task Force on Preventive Health Care guidelines, screening currently targets individuals aged 50 to 74 years at average risk of developing CRC.^2^ In response to increasing CRC incidence in younger adults and updated decision-analysis modeling,^3^ several US organizations recommend initiating screening at age 45 rather than 50 years in average-risk Americans.^4,5,6,7^ Canadian data reveal similar rising early-onset CRC rates, suggesting a potential role for expansion of screening eligibility in Canada.^2,8^

Modeling studies have demonstrated incidence and mortality benefit in screening beginning at age 50 years,^9,10^ but there is a lack of Canadian literature assessing CRC screening before age 50 years. These economic data are essential in the context of the Canadian single-payer health care system. We used OncoSim, a publicly available microsimulation tool led by the Canadian Partnership Against Cancer (https://www.partnershipagainstcancer.ca), to model the effects of earlier screening on CRC incidence, mortality, and health care costs in Canada.

## Methods

The OncoSim-CRC model is based on Canadian demographics, disease patterns, and screening/management practices.^11^ The model simulated individuals from birth to death to create a representative sample of the Canadian population. Details of the model and its validation have been described previously.^11^ We used OncoSim (version 3.4.0.3) to compare the current, guideline-recommended Canadian screening strategy (biennial fecal immunochemical test [FIT] from ages 50 to 74 years) with earlier screening (beginning at age 45 or 40 years, starting from the year 2022). To incorporate increased CRC incidence in recent birth cohorts, we adjusted the model using a similar approach to that by Peterse et al.^3^ Modeling of incidence data from the Canadian Cancer Registry was performed to estimate the cohort risk ratios (RRs) using the National Cancer Institute (NCI) Age Period Cohort Analysis Webtool.^12^ We used the 1968 to 1972 birth cohort as the reference because this was the latest cohort that would not be affected by lowering the screening age in 2022. This analysis was based on 4 birth cohorts: individuals born in 1973 to 1977, 1978 to 1982, 1983 to 1987, and 1988 to 1992, using a cohort RR of 1.17, 1.40, 1.91, and 2.29, respectively. These RRs were applied as the adenoma incidence multiplier for each cohort. Details of model parameters are outlined in eTables 1 and 2 in Supplement 1. Each scenario included 32 million simulated cases. All costs are expressed in 2019 Canadian dollars using a 0% discount rate.

Participation at first screening invitation was set to 43% based on published data.^13,14^ We carried out 2 sensitivity analyses: participation set to 60% and 43% participation with no adenoma incidence multiplier (eMethods in Supplement 1). OncoSim makes FIT screening available regardless of family history and offers screening by colonoscopy to individuals at above-average risk (eMethods in Supplement 1). Positive FIT results were followed by colonoscopy and surveillance as previously outlined.^11^

## Results

### Modeled Effect on CRC Incidence and Mortality

In this microsimulation, we observed decreased CRC incidence and mortality with earlier screening initiated at age 45 years (Figure). When results were expressed as cumulative values over 40 years (from the year a cohort’s oldest participants turn 40 years to the year where the youngest participants turn 75 years), screening beginning at age 45 years rather than 50 years yielded 12 188 fewer CRC cases and 5261 fewer CRC deaths in total among the 4 birth cohorts studied (Canadians born 1973-1992).

**Figure.**
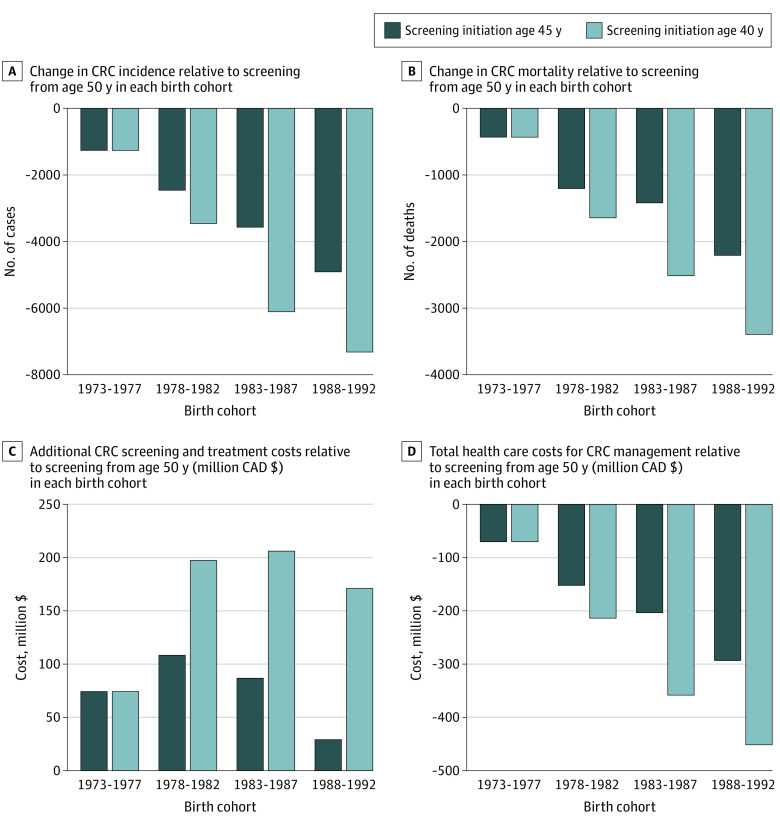
Yearly Cumulative Outcomes of Modeled Earlier Colorectal Cancer (CRC) Screening Initiation Cost of CRC management includes screening, diagnosis, treatment, management of recurrence, palliative and end-of-life care (for all CRC cases, diagnosed by screening or not). All costs are in 2019 Canadian dollars.

Modeled screening beginning at age 40 years resulted in 18 135 fewer CRC cases and 7988 fewer CRC deaths among the 4 birth cohorts over the 40-year interval compared with screening initiation at age 50 years. Both CRC incidence and mortality reductions were most pronounced in the younger birth cohorts observed (Table 1 and Figure).

**Table 1.** Cumulative Outcomes of Modeled Scenarios for CRC Screening Initiation at age 45 or 40 Years^a^^,^^b^

Measure	Birth cohort	Screening initiation age, y		
		≥50	45	40
Total cost, $ (billions)	1973-1977	7.66	7.74	7.74
	1978-1982	9.23	9.34	9.43
	1983-1987	12.34	12.43	12.55
	1988-1992	15.10	15.13	15.27
Cost of screening and diagnosis, $ (billions)	1973-1977	2.70	2.84	2.84
	1978-1982	3.04	3.30	3.46
	1983-1987	3.67	3.96	4.23
	1988-1992	4.23	4.55	4.85
Cost of CRC management, $ (billions)^c^	1973-1977	4.97	4.89	4.89
	1978-1982	6.19	6.03	5.97
	1983-1987	8.67	8.47	8.32
	1988-1992	10.87	10.58	10.42
CRC incidence	1973-1977	87 732	86 472	86 472
	1978-1982	109 472	107 014	106 015
	1983-1987	154 048	150 483	147 944
	1988-1992	192 302	187 396	184 987
CRC deaths	1973-1977	30 331	29 900	29 900
	1978-1982	37 880	36 676	36 235
	1983-1987	53 018	51 600	50 504
	1988-1992	66 568	64 361	63 171

Abbreviation: CRC, colorectal cancer.

^a^ Values are cumulative over the 40-year period from the year the oldest participants of the cohort turn age 40 years to when the youngest participants turn age 75 years.

^b^ All costs are in 2019 Canadian dollars. Screening test results included screening done by fecal immunochemical test and colonoscopy (for eligible individuals with family medical history).

^c^ Cost of CRC management included screening, diagnosis, treatment, management of recurrence, palliative and end-of-life care (for all CRC cases, diagnosed by screening or not).

### Modeled Effect on Costs

As expected, the microsimulation showed that earlier screening raised costs. Lowering the initiation age to 45 or 40 years cost an additional $298 million or $649 million, respectively, in screening and resultant treatment costs (cumulative total for all cohorts older than 40 years) (Figure). Conversely, we observed savings in the overall cost of CRC management (including costs associated with diagnosis, treatment, cancer recurrences, palliative, and end-of-life care for all CRC cases, diagnosed by screening or not) of $719 million and $1.1 billion for screening initiation at age 45 and 40 years, respectively, for all cohorts older than 40 years (Figure).

### Quality-Adjusted Life-Years (QALYs)

OncoSim calculates QALYs by multiplying a simulated individual’s years of life by a utility value based on health status. Over the 40-year period, we observed a gain of 92 112 QALYs in the 4 cohorts with screening initiation at age 45 years and 150 373 additional QALYs with screening initiation at age 40 years (Table 2). The most benefit was observed in the youngest cohort (birth years 1988-1992), where screening initiation at age 45 yielded 38 268 additional QALYs at a cost of $762 per QALY. Screening beginning at age 40 years yielded 65 305 additional QALYs in this cohort at a cost of $2622 per QALY.

**Table 2.** Quality-Adjusted Life-Years (QALYs) Gained and Additional Cost With Modeled Earlier CRC Screening Relative to Screening Initiation at Age 50 Years^a^^,^^b^

Screening initiation age, y	Birth cohort	QALYs gained	Additional cost, $ (millions)	Cost per QALY, $
45	1973-1977	8342	74	8913
	1978-1982	18 847	108	5745
	1983-1987	26 655	87	3251
	1988-1992	38 268	29	762
40	1973-1977	8342	74	8913
	1978-1982	28 192	198	7006
	1983-1987	48 534	206	4246
	1988-1992	65 305	171	2622

Abbreviation: CRC, colorectal cancer.

^a^ Values are cumulative over the 40-year period from the year the oldest participants of the cohort turn age 40 years to when the youngest participants turn age 75 years.

^b^ Cost is in 2019 Canadian dollars and includes costs associated with screening and treatment.

### Sensitivity Analyses

When screening participation was 60%, outcomes followed a similar trend to the main analysis, notably with more QALYs at a similar cost to the main scenario (eTables 3 and 4 in Supplement 1). When no adenoma incidence rate multiplier was applied, we observed a more modest benefit from earlier screening but maintained cost-effectiveness (eResults in Supplement 1).

## Discussion

In this economic evaluation computational study, OncoSim modeling showed a decrease in projected CRC incidence and mortality when screening was initiated at earlier ages, with increasing benefit in younger cohorts. Screening programs initiated at earlier ages add QALYs to this simulated population at a very modest cost per QALY compared with other life-prolonging interventions such as dialysis^15^ and decreasing cost in younger cohorts.

The main analysis (using a 43% participation rate and modified adenoma incidence by birth cohort) yielded a moderate estimate of benefits of earlier screening initiation. Sensitivity analysis using an aspirational estimate of 60% screening participation predicted increasing benefit to earlier screening if Canada is able to achieve this target (eTables 3 and 4 in Supplement 1). Sensitivity analysis using no adenoma rate multiplier predicted that earlier screening was still cost-effective even if rising CRC rates in younger generations have been overestimated (eTables 5 and 6 in Supplement 1).

Although earlier screening increased costs associated with screening and subsequent treatment, we observed overall health care costs related to CRC management to decrease. Earlier screening may identify more precancerous lesions and early-stage cancers, which are less costly to treat. However, effects on colonoscopy demand and quality, existing low rates of screening adherence, and other health system considerations need to be addressed before considering guideline changes. Although our economic data only apply directly to Canada, our findings may help justify earlier screening in other jurisdictions with similar public health care systems.

### Strengths and Limitations

OncoSim is informed by historical sources of data derived from an older screening cohort because there are currently no randomized clinical trials exploring CRC screening in younger adults, to our knowledge. The rates of participation with initial FIT, repeat screening, and compliance with follow-up colonoscopy are not known in the younger population but we estimated it to match current Canadian participation in those aged 50 years or older. Of note, although we could not control for this, a major strength of this study is that we adjusted adenoma rates by birth cohort to more accurately reflect rising rates of malignant disease in younger individuals.

## Conclusions

The findings of this economic evaluation computational modeling study suggest that lowering the screening initiation age to 45 or 40 years may reduce CRC disease burden and add life-years to the Canadian population at a cost comparable to other health care interventions.

## Notes

Supplement 1. eTable 1. OncoSim Input Parameters for FIT Screening Program eTable 2. OncoSim Input Parameters for Colonoscopy Screening Program (for Individuals with at Least One 1st Degree Relative with CRC) eMethods eTable 3. Cumulative Outcomes of Modeled Scenarios for CRC Screening Initiation at age 45 or 40 When Screening Participation Set to 60% eTable 4. Quality-Adjusted Life-Years Gained and Additional Cost with Earlier Screening Relative to Initiation at Age 50 and 60% Screening Participation eTable 5. Cumulative Outcomes of Modeled Scenarios for CRC Screening Initiation at age 45 or 40 with Screening Participation at 43% and no Adenoma Incidence Multiplier eTable 6. Quality-Adjusted Life-Years Gained and Additional Cost with Earlier Screening Relative to Initiation at Age 50 With Screening Participation at 43% and no Adenoma Incidence Multiplier eResults. Sensitivity Analyses

Supplement 2. Data Sharing Statement
