# Supplementary material for: Association of Reducing the Recommended Colorectal Cancer Screening Age With Cancer Incidence, Mortality, and Costs in Canada Using OncoSim
Source: JAMA Oncol. 2023 Jul 20;9(10):1432–6. doi: 10.1001/jamaoncol.2023.2312 (PMC10360004; doi:10.1001/jamaoncol.2023.2312)
Supplement: Supplement 1. — eTable 1. OncoSim Input Parameters for FIT Screening Program eTable 2. OncoSim Input Parameters for Colonoscopy Screening Program (for Individuals with at Least One 1st Degree Relative with CRC) eMethods eTable 3. Cumulative Outcomes of Modeled Scenarios for CRC Screening Initiation at age 45 or 40 When Screening Participation Set to 60% eTable 4. Quality-Adjusted Life-Years Gained and Additional Cost with Earlier Screening Relative to Initiation at Age 50 and 60% Screening Participation eTable 5. Cumulative Outcomes of Modeled Scenarios for CRC Screening Initiation at age 45 or 40 with Screening Participation at 43% and no Adenoma Incidence Multiplier eTable 6. Quality-Adjusted Life-Years Gained and Additional Cost with Earlier Screening Relative to Initiation at Age 50 With Screening Participation at 43% and no Adenoma Incidence Multiplier eResults. Sensitivity Analyses [file jamaoncol-e232312-s001.pdf]

Supplemental Online Content

Kalyta A, Ruan Y, Telford JJ, et al. Association of reducing the recommended colorectal cancer screening age with cancer incidence, mortality, and costs in Canada using OncoSim. *JAMA Oncol*. Published online July 20, 2023. doi:10.1001/jamaoncol.2023.2312

eTable 1. OncoSim Input Parameters for FIT Screening Program
eTable 2. OncoSim Input Parameters for Colonoscopy Screening Program (for Individuals with at Least One 1st Degree Relative with CRC)
eMethods
eTable 3. Cumulative Outcomes of Modeled Scenarios for CRC Screening Initiation at age 45 or 40 When Screening Participation Set to 60%
eTable 4. Quality-Adjusted Life-Years Gained and Additional Cost with Earlier Screening Relative to Initiation at Age 50 and 60% Screening Participation
eTable 5. Cumulative Outcomes of Modeled Scenarios for CRC Screening Initiation at age 45 or 40 with Screening Participation at 43% and no Adenoma Incidence Multiplier
eTable 6. Quality-Adjusted Life-Years Gained and Additional Cost with Earlier Screening Relative to Initiation at Age 50 With Screening Participation at 43% and no Adenoma Incidence Multiplier
eResults. Sensitivity Analyses

This supplemental material has been provided by the authors to give readers additional information about their work.

© 2023 Kalyta A, et al. *JAMA Oncol.*

eTable 1. OncoSim Input Parameters For FIT Screening Program[a]

Input Parameter	Screening initiation at age 50 (base case)	Screening initiation age lowered to 45 in 2022	Screening initiation age lowered to 40 in 2022
Screening initiation age	50 in all years	50, then 45 in 2022 and onwards	50, then 40 in 2022 and onwards
Screening cessation age	74		
FIT screening interval (years)	2		
Screening start year	2008		
Population reached by screening program based on random allocation by OncoSim (%)	100		
Participation in first screening invitation (%)	43		
Phase in of first invitation (years)	2		
Participation in repeat screens (%)	80		
Compliance to follow-up colonoscopy after positive FIT (%)	85		
FIT sensitivity (%)	30% for polyps >10 mm in size, 75% for cancer		
FIT specificity (%)	96%		
Cost of one instance of FIT (test kit + processing costs) ($)	24		
Total cost of one follow-up colonoscopy ($)	632.20		
Cost per physician visit ($)	28.55		
Cost of consultation for positive screen + polypectomy ($)	910.70		
Adenoma age rate multiplier (used to modify the "colorectal adenoma incidence rate age multiplier")	1.16 (1973-77 birth cohort)<br>1.39 (1978-82 birth cohort)<br>1.91 (1983-87 birth cohort)<br>2.29 (1988-92 birth cohort)		

Abbreviations: FIT, fecal immunochemical test.
[a] All costs in 2019 Canadian dollars.

eTable 2. OncoSim Input Parameters for Colonoscopy Screening Program (for Individuals With at Least One 1st Degree Relative With CRC)[a]

Input Parameter	Base case value
Screening initiation age	40
Screening cessation age	74
Colonoscopy screening interval (years – for individuals with family history of CRC only)	5
Screening start year	1990
Population reached by screening program based on random allocation by OncoSim (%)	50
Participation in first screening invitation (%)	100
Phase in of first invitation (years)	5 for the year 1990 then 1 for subsequent years
Participation in repeat screens (%)	80

Abbreviations: CRC, colorectal cancer.

© 2023 Kalyta A, et al. *JAMA Oncol.*

eMethods

Screening in Individuals with Above-Average Risk of CRC

OncoSim's Base Case contains a separate stream for screening in individuals with a family history of CRC (at least one first degree relative diagnosed) which we kept consistent in all scenarios. This involves screening with colonoscopy every 5 years and is made available to a random subset of 50% of individuals with family history of CRC, with 100% of invited individuals participating in a first screen: the other 50% can enter the FIT screening stream. We made no changes to this screening stream in our scenarios.

Sensitivity Analyses

In addition to the main analysis, we carried out two sensitivity analyses: one demonstrating an aspirational screening participation rate of 60% with the same adenoma rate multiplier applied to birth cohorts, and a second conservative estimate with no adenoma incidence rate multiplier and 43% participation. These two scenarios were carried out in the same way as the main analysis as outlined in eTables 1 and 2. The results of the sensitivity analyses are also expressed as cumulative values over 40 years from the year a cohort's oldest subjects turn 40 to the year the youngest turn 75.

eTable3. Cumulative Outcomes of Modeled Scenarios for CRC Screening Initiation at age 45 or 40 When Screening Participation Set to 60%[a,b]

Measure	Birth cohort	Screening initiation age		
		50	45	40
Total cost ($billions)	1973-77	7.77	7.88	7.88
	1978-82	9.28	9.44	9.56
	1983-87	12.26	12.38	12.55
	1988-92	14.88	14.92	15.11
Cost of screening and diagnosis ($billions)	1973-77	3.27	3.47	3.47
	1978-82	3.67	4.03	4.24
	1983-87	4.39	4.79	5.18
	1988-92	5.03	5.48	5.90
Cost of CRC management ($billions)[c]	1973-77	4.50	4.41	4.41
	1978-82	5.61	5.40	5.32
	1983-87	7.87	7.59	7.38
	1988-92	9.85	9.44	9.21
CRC incidence	1973-77	80,853	79,101	79,101
	1978-82	100,501	97,263	95,871
	1983-87	141,537	136,623	133,215
	1988-92	176,358	169,673	166,017
CRC deaths	1973-77	26,597	25,951	25,951
	1978-82	33,233	31,623	31,063
	1983-87	46,657	44,632	43,087
	1988-92	58,371	55,363	53,726

Abbreviations: CRC, colorectal cancer.

[a] Values are cumulative over the 40-year period from the year the oldest subjects of the cohort turn age 40 to when the youngest subjects turn age 75.

[b] All costs are in 2019 Canadian dollars. Screening test results include screening done by FIT and colonoscopy (for eligible individuals with family history).
[c] Cost of CRC management includes screening, diagnosis, treatment, management of recurrence, palliative and end-of-life care.

eTable4. Quality-Adjusted Life-Years Gained and Additional Cost With Earlier Screening Relative to Initiation at Age 50 and 60% Screening Participation[a,b]

Screening initiation age	Birth cohort	QALYs gained	Additional cost (millions)	Cost per QALY
**45**	1973-77	11,732	$107	$9,099
	1978-82	26,439	$157	$5,943
	1983-87	37,804	$118	$3,121
	1988-92	52,515	$41	$780
**40**	1973-77	11,732	$107	$9,099
	1978-82	38,329	$281	$7,333
	1983-87	68,743	$291	$4,227
	1988-92	90,710	$234	$2,574

Abbreviations: CRC, colorectal cancer; QALY, quality-adjusted life-year.
[a] Values are cumulative over the 40-year period from the year the oldest subjects of the cohort turn age 40 to when the youngest subjects turn age 75.
[b] Cost is in 2019 Canadian dollars and includes costs associated with screening and treatment.

eTable5. Cumulative Outcomes of Modeled Scenarios for CRC Screening Initiation at age 45 or 40 With Screening Participation at 43% and no Adenoma Incidence Multiplier[a, b]

Measure	Birth cohort	Screening initiation age		
		**50**	**45**	**40**
**Total cost ($billions)**	1973-77	6.92	6.99	6.99
	1978-82	7.39	7.52	7.63
	1983-87	7.82	7.96	8.15
	1988-92	8.33	8.51	8.72
**Cost of screening and diagnosis ($billions)**	1973-77	2.58	2.72	2.72
	1978-82	2.75	3.00	3.15
	1983-87	2.93	3.20	3.46
	1988-92	3.14	3.43	3.70
**Cost of CRC management ($billions)[c]**	1973-77	4.35	4.27	4.27
	1978-82	4.64	4.52	4.48
	1983-87	4.89	4.76	4.68
	1988-92	5.20	5.09	5.01
**CRC incidence**	1973-77	76,942	75,682	75,682
	1978-82	82,178	80,202	79,515
	1983-87	87,277	85,067	83,936
	1988-92	92,487	90,465	89,477
**CRC deaths**	1973-77	26,494	26,080	26,080
	1978-82	28,166	27,364	27,100

© 2023 Kalyta A, et al. *JAMA Oncol.*

	1983-87	29,763	28,804	28,252
	1988-92	31,580	30,945	30,336

Abbreviations: CRC, colorectal cancer.
[a] Values are cumulative over the 40-year period from the year the oldest subjects of the cohort turn age 40 to when the youngest subjects turn age 75.
[b] All costs are in 2019 Canadian dollars. Screening test results include screening done by FIT and colonoscopy (for eligible individuals with family history).
[c] Cost of CRC management includes screening, diagnosis, treatment, management of recurrence, palliative and end-of-life care.

eTable6. Quality-Adjusted Life-Years Gained and Additional Cost With Earlier Screening Relative to Initiation at Age 50 With Screening Participation at 43% and no Adenoma Incidence Multiplier[a,b]

Screening initiation age	Birth cohort	QALYs gained	Additional cost (millions)	Cost per QALY
**45**	1973-77	7,333	$69	$9,474
	1978-82	13,506	$130	$9,601
	1983-87	15,139	$141	$9,308
	1988-92	13,635	$178	$13,090
**40**	1973-77	7,333	$69	$9,474
	1978-82	19,351	$237	$12,227
	1983-87	26,376	$324	$12,285
	1988-92	24,038	$384	$15,995

Abbreviations: CRC, colorectal cancer; QALY, quality-adjusted life-year.
[a] Values are cumulative over the 40-year period from the year the oldest subjects of the cohort turn age 40 to when the youngest subjects turn age 75.
[b] Cost is in 2019 Canadian dollars and includes costs associated with screening and treatment.

© 2023 Kalyta A, et al. *JAMA Oncol.*

eResults: Sensitivity Analyses

Sensitivity Analyses - Effect on CRC Incidence and Mortality

Using an aspirational estimate of 60% screening participation predicts decreases in CRC incidence and mortality of greater magnitude than the main analysis using 43% participation. Screening initiation at age 45 yields 16,590 fewer CRC cases and 7,288 fewer CRC deaths compared to screening initiation at age 50 (total among the four birth cohorts studied). This represents 4,402 CRC cases and 2,027 CRC deaths avoided compared to our analysis using 43% participation.

Screening beginning at age 40 with 60% participation yields 25,046 fewer CRC cases and 11,030 fewer CRC deaths compared to initiation at age 50 (total for all birth cohorts). This is 6,911 fewer CRC cases and 3,042 fewer CRC deaths compared to the 43% participation scenario. As in the main analysis, the 60% participation scenario shows higher incidence and mortality reductions in younger birth cohorts (eTable 3).

In our conservative estimate using no adenoma rate multiplier and 43% participation, we observe more modest benefit compared to our main analysis. The model predicts 7,468 fewer CRC cases and 2,811 fewer deaths with screening beginning at age 45 and 10,274 fewer cases and 4,235 fewer deaths with screening beginning at age 40.

Sensitivity Analyses - Effect on Costs

The 60% participation scenario predicts a cumulative additional screening and treatment cost of $423 million with screening initiation at age 45 and an additional $912 million with initiation at age 40, both cumulative over 40 years and relative to screening starting at age 50. We again observe savings in the overall cost of CRC management (including cost of diagnosis, treatment, cancer recurrence, palliative and end-of-life care for all CRC cases, diagnosed by screening or not) of $999 million for screening initiation at 45 and $1.5 billion for initiation at age 40 (total for all birth cohorts over 40 years).

The conservative analysis predicts screening and treatment costs to increase by $518 million and $1.01 billion for screening beginning at age 45 and 40 respectively. Cost of CRC management is predicted to decrease by $438 and $629 million for screening beginning at age 45 and 40 respectively.

Sensitivity Analyses - Quality-Adjusted Life-Years

The 60% participation scenario predicts a total gain of 128,490 quality-adjusted life-years (QALYs) in the four birth cohorts for screening initiation at age 45 and 209,515 QALYs gained by screening from age 40 (eTable 4). Compared to 43% screening participation, this is 36,378 more QALYs for screening initiation at age 45 and 59,142 more QALYs for initiation at age 40. In the youngest birth cohort (born 1988-92) the cost per QALY is $780 and $2,574 for screening initiation at age 45 and 40 respectively, in keeping with our main analysis with 43% participation (eTable 4).

The conservative model predicts a total gain of 49,613 quality-adjusted life-years (QALYs) in the four birth cohorts for screening initiation at age 45 and 77,098 QALYs gained by screening from age 40. In the youngest birth cohort (born 1988-92) the cost per QALY is $13,090 and $15,995 for screening initiation at age 45 and 40 respectively (eTable 6). While the costs per QALY are higher in this sensitivity analysis than in scenarios using the adenoma rate multiplier, this is still cost effective compared to other accepted health care interventions.

© 2023 Kalyta A, et al. *JAMA Oncol.*
