# Supplementary material for: Association of Reducing the Recommended Colorectal Cancer Screening Age With Cancer Incidence, Mortality, and Costs in Canada Using OncoSim
Source: JAMA Oncol. 2023 Jul 20;9(10):1432–6. doi: 10.1001/jamaoncol.2023.2312 (PMC10360004; doi:10.1001/jamaoncol.2023.2312)
Supplement: Supplement 2. — Data Sharing Statement [file jamaoncol-e232312-s002.pdf]

Data Sharing Statement

Kalyta. Association of Reducing the Recommended Colorectal Cancer Screening Age With Cancer Incidence, Mortality, and Costs in Canada Using OncoSim. *JAMA Oncol*. Published July 20, 2023. doi:10.1001/jamaoncol.2023.2312

Data
Data available: No

Additional Information
Explanation for why data not available: Access to OncoSim can be requested by registering with CPAC for an account on their portal.
